# Management of urinary tract infections: what do doctors recommend and patients do? An observational study in German primary care

**DOI:** 10.1186/s12879-020-05377-w

**Published:** 2020-11-09

**Authors:** Ildikó Gágyor, Stephanie Strube-Plaschke, Katrin Rentzsch, Wolfgang Himmel

**Affiliations:** 1 Department of General Practice, Universitätsklinikum Würzburg, Josef-Schneider-Str. 2/D7, 97080 Würzburg, Germany; 2 Department of General Practice, Göttingen University Medical Center, Humboldtallee 38, 37073 Göttingen, Germany; 3 Department of Psychology, Psychologische Hochschule Berlin, Am Köllnischen Park 2, 10179 Berlin, Germany

**Keywords:** Urinary tract infection, Quality of life, Symptom assessment, Anti bacterial agents, Non-steroidal anti-inflammatory agents, Primary health care, Prospective studies, Surveys and questionnaires

## Abstract

**Background:**

Uncomplicated urinary tract infections (UTIs) in women are usually managed in primary care with antibiotics. However, many women seem to prefer to handle UTI symptoms with nonsteroidal anti-inflammatory drugs (NSAIDs) and other remedies. The aim of this study was to compare UTI management as recommended by physicians with the patients’ management at home.

**Methods:**

This prospective cohort study in German primary care is based on clinical data from local practices and patient questionnaires. Participating women completed a baseline data sheet in the practice; their urine sample was tested by a dipstick in the practice and cultured by a laboratory. The women reported treatment and symptom-related impairment on an eight-item symptom questionnaire daily for 7 days. Using growth curve models, we analysed the influence of time on the total severity score to examine how symptoms changed across days. We then examined whether symptom severity and symptom course differed between patients who took antibiotics or NSAIDs.

**Results:**

A total of 120 women (mean age of 43.3 ± 16.6 years) were enrolled. The urine dipstick was positive for leucocytes in 92%, erythrocytes in 87%, and nitrites in 23%. Physicians prescribed antibiotics for 102 (87%) women and recommended NSAIDs in 14 cases. According to the women’s reports, only 60% (72/120) took antibiotics, while the remainder took NSAIDs and other remedies. Symptoms declined from day 0 to day 6, irrespective of whether women decided to take an antibiotic, NSAIDs, none or both, as confirmed by a significant curvilinear time effect (*B* = 0.06, *SE* = 0.005, *p* < .001). The symptom course, however, was moderated by taking antibiotics so that the change in symptom severity was somewhat more pronounced in women taking antibiotics (*B* = 0.06) than in the remainder (*B* = 0.04).

**Conclusion:**

A substantial proportion of women did not follow their physicians’ treatment recommendations, and many used NSAIDs. All women had a good chance of recovery irrespective of whether they decided to take antibiotics. A sensitive listening to patient preferences in the consultation may encourage physicians to recommend and prescribe symptomatic treatment with NSAID more often than antibiotic medicines.

## Background

Uncomplicated urinary tract infections (UTIs) are common in women and are usually managed in primary care [1, 2]. Primary care physicians mainly prescribe antibiotics [3, 4], as recommended by most guidelines [5–7]. Symptomatic treatment with nonsteroidal anti-inflammatory drugs (NSAIDs) is also recommended by several guidelines for women with mild to moderate symptoms [6–8]. However, many women seem to prefer not to take antibiotics and to handle UTI symptoms with NSAIDs and other measures or remedies, presumably from their knowledge of potential harms of antibiotics and especially when they were encouraged by their physicians to delay antibiotic treatment [1, 9, 10]. In Germany, women who forego antibiotics do not have directs costs, since consultations and most of the drugs costs are covered by the health insurance [11]. While we are aware of women’s views towards UTI management, we know little about their actual management of the symptoms and to what degree their management follows the physicians’ treatment recommendations. Whether the severity of the subjective symptoms and the results of the dipstick test or urine culture play a role in women’s use of antibiotics is, to date, unknown.

The aim of the study was to compare management of uncomplicated UTI as recommended by the physicians with the patients’ management strategies at home. We were especially interested in (1) which therapies were prescribed or recommended and which treatments were used by women, (2) whether the symptoms and the urine test results influenced the therapies used by the patients and (3) whether the symptom course was different depending on the therapy used.

## Methods

### Study design

This is a prospective cohort study in the German primary care setting based on clinical data from local practices and patient questionnaires to compare the physician’s and patients’ strategies for the management of uncomplicated UTIs. The study is embedded in a clinical trial that investigates the non-inferiority of an herbal drug first and antibiotics if needed treatment approach in comparison with immediate antibiotics in women with uncomplicated UTIs. Ethics approval was obtained from the ethics committee of Göttingen Medical School (17/4/16).

### Participants

We aimed to recruit a sample of approximately 20 primary care practices in defined areas in two German federal states (Lower Saxony and Thuringia). The practices should be run by general practitioners (GPs) or community gynaecologists because both are involved in the treatment of women with uncomplicated UTIs in Germany. We sent an invitation letter to all 298 physicians in the area and offered participating physicians and nurses a honorarium (50 € and 20 € per recruited patient, respectively). All participating physicians provided written informed consent.

Over a period of 18 months, women (18 years and older) who visited the practice and had a clinical diagnosis of a UTI (based on the typical symptoms of the condition such as dysuria, urgency, frequency and low abdominal pain) were invited to participate. Women with signs of a complicated UTI (e.g., fever), symptom duration > 1 week, chronic UTI, current antibiotic therapy, UTI in the last 2 weeks, permanent catheter, anatomical abnormalities (e.g., cystic kidney), dementia, severe chronic disease, insufficient German language skills, or pregnancy were excluded. Patients were informed about the study, and those who gave informed consent received an electronic code that allowed them to access a daily electronic questionnaire for the following 7 days and were asked to complete a short telephone interview on day 28.

### Data collection

Using the information from the physicians’ electronic health records and paper-based documentation sheets, we collected the following data for each patient at inclusion:

- Dipstick results for leucocytes, erythrocytes and nitrites; results of the urine culture
- Drugs (antibiotics, whether NSAIDs were recommended, other treatment)
- Antibiotic resistance (to fosfomycin, trimethoprim, co-trimoxazole, nitrofurantoin, ciprofloxacin).

Patient-reported data included the following:

- Sociodemographic data
- Current medications (antibiotics, NSAIDs, other) at inclusion and daily until day 7
- Severity of each UTI symptom was assessed with a self-report questionnaire, the UTI-SIQ-8 Questionnaire. The questionnaire consists of 4 items to assess the symptom severity for dysuria, urgency, frequency and low abdominal pain, scored from 1 (no symptoms at all) to 5 (very strong symptoms) and 4 items to assess the impairment of activity by these symptoms, scored from 1 (no impairment at all) to 5 (very strong impairment).
- Data on recurrent UTI, pyelonephritis.

### Study procedures

After inclusion, all participating women completed a baseline data sheet in the practice. The urine sample of each patient was tested by a dipstick in the practice and cultured by a central laboratory. The cut-off value for a positive urine culture was > 10^2^.

The study centre was informed about each new included patient by fax and email and informed the patients how to access a special website with a personal code where they could fill in a questionnaire each day. The patients received either a text message or an email on each of the following 6 days that reminded them to complete the questionnaire.

### Sample size

One aim of the larger project [12] was to validate a new symptom questionnaire that we planned to use for this trial, especially its sensitivity to change. To accomplish this aim, we calculated a sample size of 250 patients to detect a medium effect with a power of 85% using latent variables. Since it proved difficult to reach such a sample size in busy primary care practices, we made use of multilevel models on basis of a high number of data entries and manifest variables in the present article, which ensures the statistical power of our analyses to investigate the symptom course. The questionnaire’s sensitivity to change will be thoroughly studied, and the results will be published elsewhere.

### Statistical analysis

Baseline data for the clinical and patient characteristics and the dipstick and urine culture results were first analysed descriptively. We compared the number of women taking antibiotics, as reported in the practice documentation and reported by the women themselves. We calculated both the single scores of the 8-item questionnaire and the mean of the 8 items as a total UTI severity score (“total score”) for each day. The total score ranged from 1 (no symptoms/impairment at all) to 5 (very strong symptoms/impairment).

We then investigated the change in symptoms across days. Because we did not reach our planned sample size for latent variable analyses, we used growth curve models based on multilevel modelling with manifest variables [13] with the R package lme4 [14]. These analyses are useful for handling nested data structures, for example, repeated measures nested within participants, and can detect significant trends. In the present study, daily reports about symptoms (level 1) were nested within patients (level 2). This allowed us to investigate within-person effects—i.e., how symptoms change across days—and between-person effects—i.e., whether symptoms at baseline differ between women who took antibiotics or NSAIDs and those who did not, and how the symptom course differs between patients who did or did not take antibiotics or NSAIDs.

First, we analysed the influence of time (days) on the total severity score to examine how symptoms changed across days. In this model, we controlled for the influence of taking antibiotics or NSAIDs to investigate whether there was a decline in symptoms over time irrespective of taking antibiotics or NSAIDs.

Two further models were run to examine the impact of taking antibiotics and taking NSAIDs (both level 2 predictors), as well as time (level 1 predictor) and the respective cross-level interactions on the outcome variable symptom, severity. For all analyses, we included a linear and a quadratic effect of time to model the decline in symptoms as curvilinear. The intercepts and slopes of the time variables were modelled as random.

## Results

### Clinical and patient characteristics

A total of 18 practices took part in the study, and 131 women were included. We excluded 11 patients due to screening failure or technical reasons, such as missing or erroneous data, resulting in a valid sample of 120 women with a total of 769 symptom reports across 7 days of measurement. The women’s mean age was 43.3 ± 16.6 years; 62% of them were employed, 77% lived in a partnership, 68% had children, 16% were pupils, 6% were homemakers and 13% were retired. One-third (38/118) reported that the symptoms lasted no longer than 2 days, another third (42/118) had symptoms for 3 to 5 days, while 98 (82%) expected to have a UTI, and 25% felt feverish.

### Dipstick test and urine culture results

The urine dipstick test was performed for all participating women and was positive for leucocytes in 92% (108/118), for erythrocytes in 87% (103/118) and for nitrites in 23% (26/111) (Table 1); 82% (96/118) of the women had positive urine culture. *E. coli* was found in 78% (74/96) of the positive urine cultures. Other bacteria were *Enterococcus faecalis* in 8% (8/96), *Staphylococcus saprophyticus* in 5% (5/96), and *Proteus mirabilis* and *Acinetobacter baumanii* in 2% (2/96) each. The resistence of *E. coli* was as follows: fosfomycin: 0%, nitrofurantoin: 1%, ciprofloxacin: 6%, and cotrimoxazole: 19%. Of those women who suspected a UTI, 81% (79/97) had a positive urine culture (data for 1 woman was missing).

**Table 1:** Test results and drug intake; n (%)

Test results	Taking antibiotics^a^				All^b^		P***
	Yes		No				
Leucocytes							0.17
Positive	67	(62.0)	41	(38.0)	108	(91.5)	
Negative	4	(40.0)	6	(60.0)	10	(8.5)	
Erythrocytes							0.25
Positive	64	(62.1)	39	(37.9)	103	(87.3)	
Negative	7	(46.7)	8	(53.3)	15	(12.7)	
Nitrites							0.34
Positive	18	(69.2)	8	(30.8)	26	(23.4)	
Negative	50	(58.8)	35	(41.2)	85	(76.6)	
Urine culture^c^							0.49
Positive	60	(62.5)	36	(37.5)	96	(81.4)	
Negative	12	(54.6)	10	(45.4)	22	(18.6)	

^a^According to patient reports

^b^Percentages in this column comparing positive vs negative results

^c^Bacterial count > 10^2^ cfu/mL

***Chi^2^-test for the association between test results and the women’s decision whether or not to take antibiotics

### Physician and patient management of UTI

Physicians prescribed antibiotics to 102 (87%) of the participating 120 women, most often fosfomycin (55%), cotrimoxazole (11%), nitrofurantoin (9%), ciprofloxacin (9%), and cefuroxime (7%). According to the women’s reports, 37 (31%) took antibiotics alone, 15 (13% took NSAIDs alone, 35% (30%) took both and 31 (26%) took neither. In other words, only 60% (72/120) of the patients reported having taken antibiotics. While the physicians recommended additional treatment with NSAIDS to only 14 women, 51 (43%) women decided to take NSAIDs; 35 (49%) of those who took NSAIDS also took antibiotics, and 16 (33%) did not take antibiotics.

### Associations among treatment, symptom course and urine test results

Table 2 shows the symptom course, as perceived by the women, divided into those who took antibiotics and those who did not. Of all symptoms, the frequency and urgency of micturition was perceived as the strongest on day 0, with a score of 3.5 each reported by women taking antibiotics, and 3.2 or 3.1, respectively by the remainder (Table 2). All symptoms declined over the next days and were below 2.0 (= “moderate”) on day 3. This tendency can also be seen in the total score, the mean of all 8 items, which sharply fell from 3.0 (antibiotic drugs) or 2.7 (no antibiotic drugs) on day 0 to 1.9 or 1.7, respectively on day 2 and below 1.7 on day 3.

**Table 2:** Symptom severity across days

Symptom / impairment^a^	Day; m (SD)						
	0	1	2	3	4	5	6
Urgency							
AB^b^	3.5 (1.0)	2.7 (1.0)	2.3 (0.9)	1.9 (0.9)	1.7 (0.8)	1.7 (0.9)	1.6 (0.8)
Non-AB^c^	3.2 (1.2)	2.2 (1.0)	2.1 (1.0)	1.8 (0.8)	1.7 (0.7)	1.6 (0.8)	1.5 (0.8)
Dysuria							
AB	3.0 (1.3)	2.3 (1.2)	1.8 (0.9)	1.5 (0.7)	1.3 (0.6)	1.4 (0.8)	1.3 (0.6)
Non-AB	2.4 (1.3)	1.9 (0.8)	1.6 (0.7)	1.5 (0.7)	1.4 (0.7)	1.4 (0.8)	1.4 (0.6)
Frequency							
AB	3.5 (1.0)	2.6 (1.0)	2.1 (0.9)	1.8 (0.9)	1.6 (0.9)	1.6 (0.8)	1.4 (0.7)
Non-AB	3.1 (1.2)	2.2 (0.9)	2.0 (1.0)	1.7 (0.8)	1.5 (0.7)	1.5 (0.7)	1.6 (0.7)
Lower abdominal pain							
AB	2.4 (1.1)	2.0 (1.0)	1.6 (0.6)	1.5 (0.7)	1.3 (0.6)	1.3 (0.6)	1.2 (0.5)
VNon-AB	2.7 (1.2)	2.1 (1.1)	1.9 (0.9)	1.6 (0.8)	1.6 (0.7)	1.5 (0.9)	1.4 (0.7)
Impairment due to urgency							
AB	3.0 (1.1)	2.3 (1.0)	2.0 (1.0)	1.6 (0.8)	1.5 (0.7)	1.4 (0.7)	1.4 (0.7)
Non-AB	2.8 (1.2)	2.0 (1.1)	1.7 (0.8)	1.5 (0.7)	1.4 (0.7)	1.5 (0.7)	1.3 (0.6)
Impairment due to dysuria							
AB	2.8 (1.3)	2.2 (1.1)	1.7 (0.8)	1.4 (0.6)	1.3 (0.6)	1.3 (0.7)	1.3 (0.6)
Non-AB	2.3 (1.2)	1.7 (0.9)	1.5 (0.7)	1.4 (0.7)	1.4 (0.7)	1.4 (0.8)	1.3 (0.6)
Impairment due to frequency							
AB	3.1 (1.2)	2.3 (1.0)	1.8 (0.9)	1.6 (0.8)	1.4 (0.7)	1.4 (0.7)	1.3 (0.7)
Non-AB	2.7 (1.2)	2.0 (0.9)	1.7 (0.8)	1.5 (0.7)	1.4 (0.6)	1.5 (0.8)	1.5 (0.7)
Impairment due to pain							
AB	2.3 (1.1)	1.9 (1.0)	1.6 (0.7)	1.4 (0.7)	1.2 (0.6)	1.2 (0.6)	1.2 (0.4)
Non-AB	2.5 (1.2)	2.0 (1.0)	1.6 (0.7)	1.5 (0.7)	1.5 (0.7)	1.5 (0.8)	1.4 (0.7)
Total score							
AB	3.0 (0.8)	2.3 (0.8)	1.9 (0.7)	1.6 (0.6)	1.4 (0.6)	1.4 (0.6)	1.3 (0.5)
Non-AB	2.7 (0.9)	2.0 (0.8)	1.7 (0.6)	1.6 (0.6)	1.5 (0.6)	1.5 (0.7)	1.4 (0.6)

^a^Symptom severity was assessed from 1 (no symptoms at all) to 5 (very strong symptoms) and impairment of activity by these symptoms from 1 (no impairment at all) to 5 (very strong impairment)

^b^Women taking antibiotic drugs

^c^Women not taking antibiotic drugs

Based on multilevel models, the difference in symptom severity at baseline between women who decided to take antibiotics and those who did not was not significant (*B* = 0.36, *SE* = 0.20, *p* = .08). Women who decided to take NSAIDs had a somewhat higher baseline score compared to the remainder (*B* = 0.43, *SE* = 0.16, *p* = .01).

**Fig. 1:**
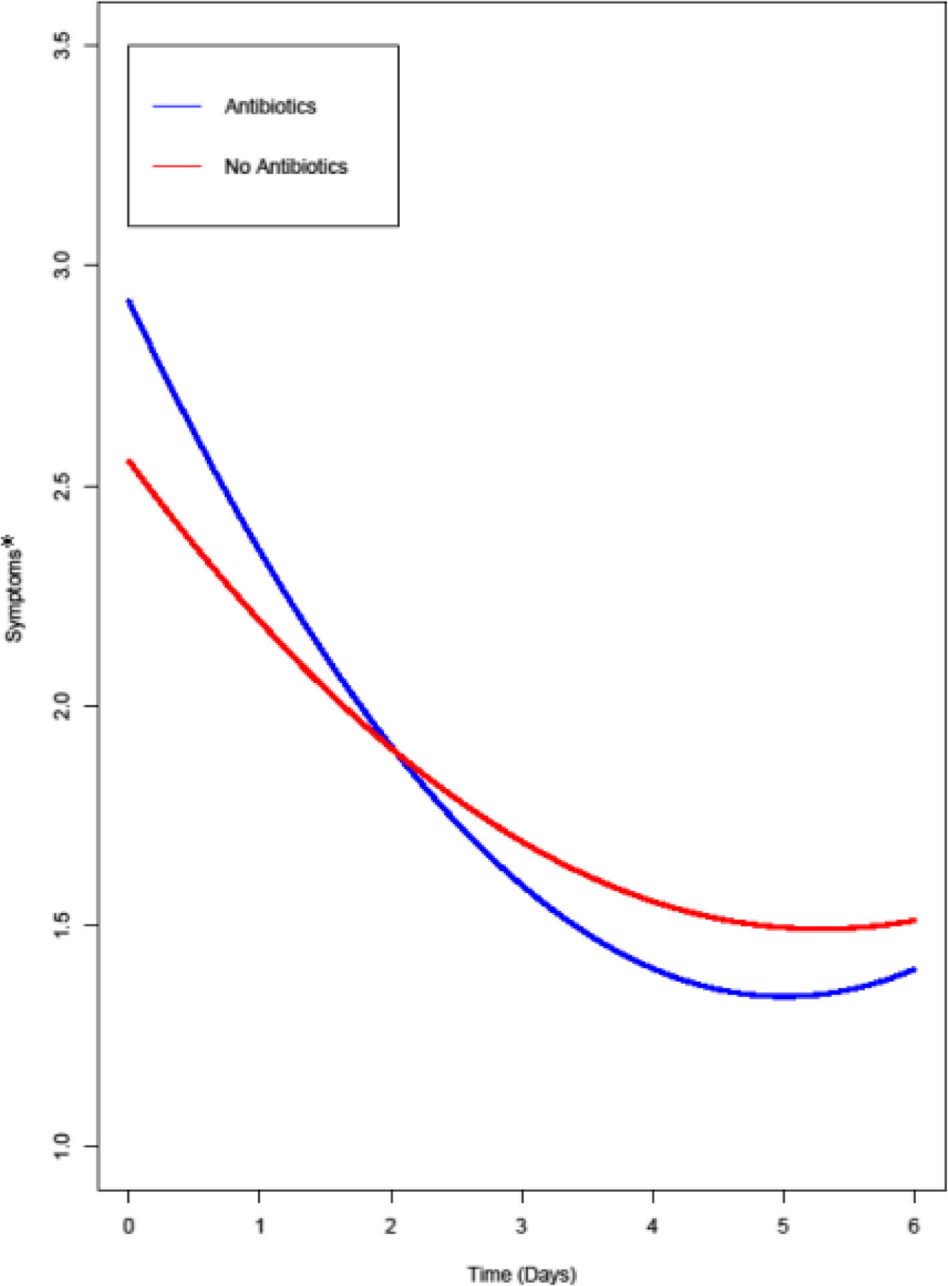
Change in symptom severity for patients taking or not taking antibiotics. * Mean total score, ranging from 1 (no symptoms/impairment at all) to 5 (very strong symptoms/impairment); all values are predicted values, based on growth curve modeling

Symptoms declined from day 0 to day 6, irrespective of whether women decided to take an antibiotic, NSAIDs, none or both, as confirmed by a significant curvilinear time effect (*B* = 0.06, *SE* = 0.005, *p* < .001). The symptom course, however, was moderated by taking antibiotics (Fig. 1). The results revealed a significant cross-level interaction between the change in symptoms and antibiotics (*B* = 0.02, *SE* = 0.01, *p* = .03), whereas there was no such effect for taking NSAIDs (*B* = 0.001, *SE* = 0.01, *p* = .92). This can also be seen in Fig. 1, where the change in symptom severity was more pronounced in women taking antibiotics (B = 0.06) than in women who refrained from taking antibiotics (*B* = 0.04).

We found no strong associations between the dipstick test and the women’s decision to take an antibiotic. Of those women with a positive test for leucocytes, 62% (67/108) took an antibiotic; of those with a negative test for leucocytes, 40% (4/10) took an antibiotic (Table 1). The associations were weaker in the case of erythrocytes and nitrites.

There was also no strong association between the urine culture results, as later reported by the laboratory, and the treatment strategy (antibiotic or symptomatic) women decided to follow. Of those women with a confirmed UTI (positive urine culture), 63% took an antibiotic, while 55% of those with a negative result also took an antibiotic (Table 1). The respective figures for NSAIDs were 42% (40/96) in the case of a positive urine culture and 45% (10/22) in the case of a negative result.

A re-occurrence of a UTI during the follow-up period was reported by 13/103 (13%;) patients on day 28 at the telephone interview (data were missing for 13 women); of those who took antibiotics at inclusion (*n* = 65), it was 6 women (9%) and of those who did not (*n* = 38), it was 7 women (18%); 12 (92%) of the 13 women with a re-occurence had received an antibiotic prescription from their physicians. One case of pyelonephritis occurred in a woman who was treated with fosfomycin at inclusion. This woman was treated with cefuroxim.

## Discussion

The results of this prospective cohort study show that a substantial proportion of women did not follow their physicians’ recommendation for antibiotic treatment and many of them used NSAIDs, although NSAIDs were rarely prescribed or recommended by their physicians. Dipstick results, urine culture results and symptom severity were not strongly associated with women’s decisions for or against the recommended treatment. Only the decision to take NSAIDs was significantly associated with the symptom severity at baseline. The UTI symptoms significantly declined across days, irrespective of whether women decided to take an antibiotic, NSAIDs, none or both. However, we detected a significant cross-level interaction between the change in symptoms and use of antibiotics, meaning that the change in symptom severity was somewhat more pronounced in women taking antibiotics than in the remainder.

### Strengths and limitations

The study provides information about management of uncomplicated UTI in primary care beginning with the consultation, investigations and tests and proceeding to the physicians’ treatment decisions and the women’s actual management strategies at home, including interactions with and outcomes of the symptom course.

Although the number of participating women was rather small, the data suggest that the study population was representative compared with other UTI studies in Germany. Baseline data, such as the proportion of patients with a positive urine culture (approx. 75%) [15–18], the proportion of patients with *E. coli* infections (ca 75%) and the susceptibility data, were comparable with the results of other observational studies in Germany [19, 20]. Most importantly, the sample was large enough to investigate longitudinal changes in symptom severity across days with high statistical power, especially when using multilevel modelling on basis of 769 reports within person and 120 units between person. Finally, since we found a significant decline in symptom severity across days and, given the number of patients and data reports, we can conclude with high statistical power that women had a good chance of recovery irrespective of whether they decided to take antibiotics.

In contrast, an observational study is not adequate to compare the outcomes of the two treatment approaches. Therefore, we cannot conclude that a symptomatic approach is more or less equivalent to antibiotic treatment. In a previous trial we could demonstrate a better treatment success of fosfomycin in terms of symptom burden compared with ibuprofen (15).

Although the safety of the symptomatic treatment approach was not a main focus of the study, we should emphasize that only one case of pyelonephritis occurred in a woman who was treated with antibiotics first and that only 13 women had a second episode of a UTI in the following 3 weeks, nearly half of whom had been treated with antibiotics.

The observational study allowed us to investigate the daily symptoms of patients with uncomplicated UTIs. Growth curve modelling allows the estimation of different growth patterns and the estimation of inter-individual differences in intra-individual change over time, and it is more robust to violations of assumptions than, for example, repeated-measures ANOVA.

### Comparison with existing literature

Previous research investigated both the physicians’ treatment approach [21–23] and the patients’ management of UTIs [3, 10, 24]. These studies provided data about tests used in local practices, the susceptibility and resistance of UTI bacteria and/or physicians’ guideline adherence [25–27]. In patient-focused studies, variations in symptom presentation or patient views of the reason for their infection have been investigated. Our study sheds light on whether the decisions made in the consultation were implemented by patients at home, illuminating the gap in the doctor-patient interaction.

Although not all women were prescribed antibiotics, the rate of antibiotic prescriptions was rather high (87%), but it is in accordance with data from other countries [25, 28], for example, an antibiotic prescription rate of 82% in a recent Hong Kong study in primary care, and data from a Spanish study with an even higher proportion of antibiotic treatment (96%).

In several randomized controlled trials [15–18], the symptom course of UTIs, usually assessed and documented by the women themselves, was compared between those immediately prescribed antibiotics and those prescribed symptomatic treatment. The symptoms mostly resolved in both groups, with a somewhat longer duration with symptomatic treatment. Similar to the results of these RCTs, we also found that the women taking antibiotics recoverd somewhat faster. No less remarkable is the fact that women who did not take antibiotics also reported a rapid decline of the UTI symptoms, as also found by Little et al. [29] under standardized conditions.

We found only moderate, non-significant associations between dipstick results, which were immediately available at the consultation and the women’s decision whether to take antibiotics. There was no association between the results of the urine culture, available several days after the first consultation, and the initial decision to take antibiotics. On first view, this result may be surprising because one might have supposed that their decision intuitively follows the ‘real biochemical facts’. This is obviously too simplistic a view that reduces patients to their disease, as Di Paleo et al. [30] suggested it in their review of personalized medicine; rather, the women seem to balance the invasive character of an antibiotic drug against the severity of symptoms.

### Implications for practice

This study is another plea for patient participation and shared decision making to form key parts of patient-centred care [31], this time in the case of uncomplicated UTIs. Women seem to know the best treatment approach to manage their UTI symptoms. Sensitive listening to patient preferences in the consultation may encourage physicians to recommend and prescribe symptomatic treatment more often than antibiotic medicines. However, GPs who prefer to delay antibiotic treatment are sometimes frustrated with patients who expect to get well quickly with antibiotics. They are faced with a complex diversity of factors influencing the culture of antibiotic prescribing, as described in an Irish study [32], and have to accept that the path to prudent prescribing is long and strenuous. In this respect, studies such as ours may provide physicians with arguments that can motivate and support more women in choosing a symptomatic treatment, at least initially.

Following the principles of medicines optimization of the National Institute for Health and Care Excellence (NICE) [33], physicians could help women by discussing their preferences and what is important to them about managing their condition and their medicines and recognize and accept that the women’s values and preferences may be different from their own.

Doctors should understand that women’s disease management will be affected by individual preferences for particular treatment modalities, the avoidance of certain side effects and a personal benefit-harm trade-off analysis of the available interventions and may differ in the level of priority they give to health and symptom recovery compared to other problems [30]. In the end, their decisions seem to have been wise because those who decided to take only NSAIDs fared nearly as well as those who took antibiotics.

## Conclusions

Women with uncomplicated UTIs clearly know best what they need and have a good chance of recovery irrespective of whether they decide to take antibiotics. The need to alleviate UTI symptoms with NSAIDS should be considered in treatment recommendations. When physicians are aware of this fact, they may feel encouraged to recommend and prescribe symptomatic treatment more often than antibiotics.

## Data Availability

The data used for the current study are available from the corresponding author on reasonable request.

## Abbreviations

UTI: Urinary tract infection
NSAIDs: Nonsteroidal anti-inflammatory drugs
GP: General practitioner
NICE: National Institute for Health and Care Excellence (United Kingdom)
